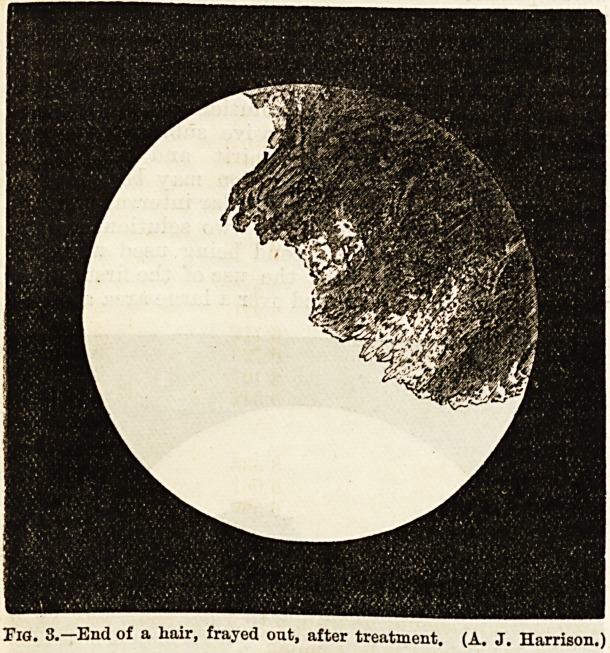# The Treatment of Ringworm at Bristol General Hospital and Elsewhere

**Published:** 1893-10-21

**Authors:** 


### THE TREATMENT OF RINGWORM AT BRISTOL GENERAL HOSPITAL, AND ELSEWHERE.

Dr. Harrison's method has now been in use some seven years, and is probably one of the simplest and most efficient yet discovered. With reasonable care most cases of ringworm can be cured by it, but precautions against reinfection are needed if relapses are to be avoided. The hair is kept short, but not shaved, and glycerine of carbolic acid is applied to the parts of the scalp which are not undergoing treatment. Contaminated clothes, bedding, and furniture may prevent the success of any method. The essence of Dr. Harrison's plan is to soften the hairs by potash, and then to take advantage of their permeable condition to soak them through and through with iodide of potash and mercuric chloride, so as to produce the biniodide in their tissues, just as in Dr. Jamieson's method we get sulphurous acid. To most solvents hair is singularly resistant, and the choice of these substances was the outcome of a series of physical experiments. If the head has been undergoing other treatment, the skin should be allowed to get sound, after which the worst patches are washed with a first solution containing liquor potassæ and spirits of wine in equal parts and half a drachm of iodide of potash to the ounce. This is done two or three times at intervals of a few days. While the hair is still just damp from the last dressing the second solution is applied, containing four grains of corrosive sublimate to the ounce in equal parts of spirit and water. A second painting with this solution may be given in two days time, and a third at a similar interval. In the next and subsequent weeks the two solutions are applied every few days, the second being used when the scalp has nearly dried after the use of the first. The washes should not be applied over a large area at once,

Fig. 2.—A hair partly twisted and broken. Very few conidia after one month's treatment. (A. J. Harrison.)

but a small section should be first got under, and the rest taken by degrees. Still, with reasonable care, there should be no risk of mercurial poisoning, and if the skin is in any way irritated, weaker solutions must be employed. A modification has been introduced for out-patients and home use, which has great advantages from its simplicity and harmlessness, and which has shown its value in a long course of trial. While the action of the caustic alkali is retained, and carbolic acid is used as the parasiticide, the two washes are replaced by an ointment which is rubbed into the patch twice a day, and allowed to remain on. The formula for the ointment is as follows: Caustic potash gr. ix., carbolic acid gr. xxiv., lanoline and oil of cocoanut of each ʒss., to be well rubbed together. S.—A small portion to be rubbed in night and morning. The hair, as in the former plan, is cut short, but not shaved, and the treatment is persistently carried out for a period of from one to three months. The healthy parts of the scalp may be dressed with a pomade containing boric acid ointment and eucalyptus ointment, of each two parts in six. This may be used as a preventative on the heads of the persons with whom the child lives. There is generally improvement after two or three weeks treatment, the conidia are fewer in number, and the bare patches begin to show new hairs. The accompanying photographs are taken from cases at different stages of treatment by the ointment. The first shows the state when the patient was first seen, the hair being crowded with spores.. From the same patient, but a month later, the second one was taken. The diminution in the numbers of the parasites is very marked, but the structure of the hair is partly destroyed in the usual manner. The third shows a damaged hair from

Fig. 3.—End of a hair, frayed out, after treatment. (A. J. Harrison.)

another case where the disease had almost entirely disappeared after six weeks dressing, and a complete cure followed. We are indebted to Dr. Harrison for allowing us to reproduce these engravings from a former paper read before the British Medical Association in 1888, and published in the journal for 1889. Both methods have been in constant use since then with most satisfactory results. Under any remedy much care is required to ensure constant regular dressing of every patch. Finally, hairs should be tested after the treatment has been carried on some time to discover the presence of spores. This may be done under the microscope, or by cultivation, or even by their turning white, if diseased, when dipped in chloroform. The advantage of cultivation on a faintly acid solution of dextrose is that a score of hairs may be dropped on to the medium together, and a growth existing in any one of them will be detected without more trouble.

Whatever treatment we adopt it must be continued until no trace of the disease remains, and until no recurrence is seen after many weeks' rest. Meanwhile children may be even allowed at school if close caps are worn, and the hair cut short, and dressed with strong boric solution or ointment, and washed with soft soap frequently. Practically, however, caps are thrown off in play, and dresses become infected so that isolation is on the whole safer.

It will be seen that all these plans of treatment aim at a general disinfection of the surface, combined with powerful remedies directed to the worst patches. These may be simple antiseptics, or may act by depriving the the plant of the water and oxygen necessary for its life. Possibly some antiseptic like alumnol, which is not precipitated by an excess of albumen, might well be used in combination with other remedies to disinfect the deeper layers of the skin, after the hairs have been thoroughly purged by Dr. Harrison's methods. We would suggest that that plan is thoroughly efficacious, as to the germs in the hairs themselves, but the real difficulty under every method is to reach those which ramify in the lower layers of the epidermis in a more or less albuminoid medium.

## Figures and Tables

**Fig. 2. f1:**
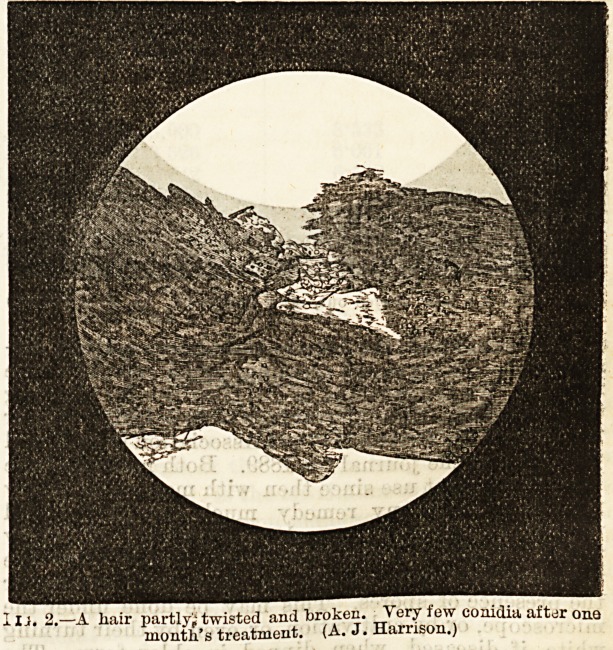


**Fig. 3. f2:**